# Association of microRNA-146a and their target gene IRAK-1 polymorphism with enthesitis related arthritis category of juvenile idiopathic arthritis

**DOI:** 10.1186/1755-8166-7-S1-P69

**Published:** 2014-01-21

**Authors:** Sushma Singh, Geeta Rai, Amita Aggarwal

**Affiliations:** 1 Department of Clinical Immunology, Sanjay Gandhi Post Graduate Institute of Medical Sciences, Lucknow, India; 2 Department of Molecular and Human Genetics, Faculty of Science, Banaras Hindu University, Varanasi, India

**Keywords:** MicroRNAs, miR-146a, rs2910164, Polymorphisms, JIA-ERA

## Background

MicroRNAs (miRNAs) are non-coding RNA molecules that play pivotal role in modulating the expression of multiple target genes at the post-transcriptional level. Single Nucleotide Polymorphisms (SNP) in pre-miRNAs can alter miRNA expression, and polymorphism in target molecules can affect binding to target mRNA. Studies have shown an association between miR-146a polymorphisms and autoimmune disease. Taking into account that interleukin-1 receptor-associated kinase-1 (IRAK-1) is a target of miR-146a, we studied the association between SNPs of miRNA-146 and its target IRAK-1 with susceptibility to Juvenile Idiopathic Arthritis-Enthesitis Related Arthritis (JIA-ERA).

## Methods

One hundred and fifty patients with JIA-ERA (ILAR criteria) were included in the study. 216 blood donors (201 male) with a mean age of 30.5 years served as controls. miR-146a (C/G) (rs2910164) and it’s target IRAK-1(C/T) (rs1059703) at Exon 12 region and IRAK-1 (A/C) (rs3027898) at 3’UTR polymorphisms were analyzed using PCR-RFLP method.

## Results

Among 150 patients, 134 were males and the mean age at onset of disease was 11 (4-16) years, mean disease duration was 4.5 (0.3-12) years. 22 had uveitis and 21 had positive family history. 73 had enthesitis and 75 had inflammatory back pain and all had arthritis. 116 were HLA B27 positive.

Genotype frequency of miR-146a gene was in Hardy Weinberg equilibrium in healthy controls whereas in IRAK-1 genotype the frequency was contrary owing to its presence on chromosome X.

The genotype frequency for miR-146a were different in controls and patients [GG (51.85% vs 50.0%), GC (42.13% vs 37.29%) and CC (6.02% vs 12.71%), OR = 2.18; 95% CI 1.02 – 4.68; p value = 0.0418]

IRAK-1 (rs1059703) allelic frequencies in controls and patients were similar [CC (33.8% vs 32.9%), and TT (66.2% vs 67.0%)]. IRAK-1 (rs3027898) allelic frequencies were also similar among control and patients [CC (70.1% vs 76.1%) and AA (29.8% vs 23.9%)].

## Conclusion

The CC genotype of the miR-146a rs2910164 polymorphism was significantly associated with the susceptibility to JIA-ERA.

